# Management Patterns of Teduglutide Use in Short Bowel Syndrome: A Survey of 70 Healthcare Professionals

**DOI:** 10.3390/nu16213762

**Published:** 2024-11-01

**Authors:** Felix Harpain, Slobodan Milicevic, Lucy Howard, Patricia Biedermann, Ulrich-Frank Pape

**Affiliations:** 1 Department of General Surgery, Division of Visceral Surgery, Medical University of Vienna, 1090 Vienna, Austria; 2 Takeda Pharmaceuticals International AG, 8152 Glattpark, Switzerland; 3 Adelphi Real World, Bollington SK10 5JB, UK; 4 Department of Hepatology and Gastroenterology, Campus Charité Mitte and Campus Virchow-Klinikum, Charité Universitätsmedizin Berlin, 13353 Berlin, Germany; ul.pape@asklepios.com; 5 Department of Internal Medicine and Gastroenterology, Asklepios Klinik St. Georg, Asklepios Medical School, 20099 Hamburg, Germany

**Keywords:** short bowel syndrome, intestinal failure, teduglutide, parenteral nutrition, survey

## Abstract

Background: This study aimed to gain real-world insights from healthcare professionals (HCPs) regarding the management of adult patients with short bowel syndrome and intestinal failure (SBS-IF) who received teduglutide and achieved parenteral support (PS) independence or PS volume stability for ≥12 months. Methods: This cross-sectional survey was conducted in five European countries and Canada via a self-reported questionnaire (November 2022–March 2023) among HCPs who manage patients with SBS-IF and who had prescribed teduglutide to ≥5 patients with SBS-IF receiving PS. Results: Of the 70 HCPs who completed the survey, almost all reported managing patients with SBS-IF who achieved PS independence or PS volume stability (99%, 69/70 and 97%, 68/70, respectively) and maintained the standard teduglutide dose, without changes. A total of 52 HCPs managed patients who achieved PS independence and discontinued teduglutide. Of these HCPs, 73% (38/52) anticipated that these patients would remain PS-independent, not requiring PS reintroduction. Of the remainder, 79% (11/14) estimated that ≤40% of these patients would require PS reintroduction. While many HCPs (81%, 42/52) would reintroduce teduglutide in patients who discontinued its use after achieving PS independence, none would do so for patients who discontinued teduglutide after achieving PS volume stability if a patient’s condition worsened. Conclusions: This survey found that patients with SBS-IF can achieve PS independence or PS volume stability with teduglutide treatment. However, some HCPs (27%, 14/52) believe that a proportion of patients discontinuing teduglutide after achieving PS independence will require PS reintroduction. This survey suggests that teduglutide treatment should continue uninterrupted, unless clinically indicated, but this requires confirmation in future studies.

## 1. Introduction

Short bowel syndrome (SBS) is a rare, malabsorptive disorder typically caused by extensive surgical resection of the intestinal tract [1,2,3]. Patients with SBS may develop varying degrees of chronic intestinal failure (IF) (defined as the decrease in gut function below the minimum necessary for the absorption of nutrients and/or water and electrolytes), such that intravenous administration of nutrients is required to provide nutritional support [1,4]. In patients experiencing irreversible IF, long-term parenteral support (PS), i.e., parenteral nutrition (PN) and/or intravenous fluid and electrolyte administration, is required to provide macronutrients, water, and electrolytes [1,2,4,5]. However, IF with long-term reliance on PS can lead to life-threatening complications, such as liver disease and sepsis, reduced patient quality of life (QoL), and significantly increased mortality [5,6,7,8,9]. Therefore, therapies that decrease reliance on PS are of considerable importance for the treatment and management of patients with SBS-IF.

Teduglutide, a glucagon-like peptide-2 (GLP-2) analog, is a hormonal therapy for SBS-IF that promotes intestinal mucosal growth, thereby improving nutrient absorption and reducing the need for patients with IF to rely on PS [10,11,12,13,14,15,16]. Teduglutide is approved in Argentina, Australia, Brazil, Canada, Chile, Columbia, Europe, Japan, and the USA at 0.05 mg/kg once daily (standard dose) for the treatment of adult and pediatric patients with SBS who depend on PS [17,18,19,20,21]. In placebo-controlled clinical trials, teduglutide promoted changes in intestinal structure and improved intestinal absorption, thereby reducing the volume and number of days of PS required by patients with SBS-IF [10,12,13,14,15]. In France, a retrospective multicenter study (*N* = 54 patients) showed that most (85%) adult patients with SBS-IF treated with teduglutide for ≥6 months were able to reduce the volume of PS received by ≥20% at 24 weeks, and 24% were completely weaned off at this point [22]. Furthermore, a systematic literature review (SLR) (10 studies, *N* = 470 patients) found that the response rate (defined as a ≥20% reduction in PS) to teduglutide among patients dependent on PS increased to 82% after 2 years or longer, compared with 64% at 6 months and 77% at 1 year [1]. Although real-world evidence in patients with SBS-IF who have been weaned off PS is limited, data suggest that teduglutide shows high efficacy with reasonable tolerability [2,3,4,22,23,24,25,26,27,28,29,30,31].

The management of patients with SBS-IF requires a multidisciplinary team with a patient-tailored treatment approach comprising different specialist healthcare professionals (HCPs) who coordinate dietary, fluid, and pharmacological care; manage comorbid complications; and evaluate the benefit–risk ratio of teduglutide treatment [32,33,34]. To ensure the optimal use of teduglutide, HCPs require support and guidance; however, few guidelines exist regarding the use of GLP-2 analog treatment in patients with SBS-IF [33,35]. To our knowledge, there is a lack of information on how clinical guidelines for teduglutide use [33,35] are implemented and whether these guidelines influence real-world decision making regarding teduglutide use.

This study aimed to gain real-world insights into the management of patients with SBS-IF from HCPs experienced in managing patients with SBS-IF via the use of teduglutide, with a focus on those who achieve PS independence or PS volume stability.

## 2. Materials and Methods

### 2.1. Study Design and Participants

A cross-sectional survey was conducted in six countries (Austria, Canada, France, Germany, Italy, and Spain) to gain insights from HCPs responsible for treatment decisions regarding and/or managing patients with SBS-IF who require PS, and who prescribe teduglutide when clinically appropriate. A one-time (single), self-reported, and self-completed online questionnaire (~30–45 min completion time; the HCP Survey is available in the Supplementary Materials) was conducted to collect data on HCP perceptions regarding the treatment and outcomes of their patients with SBS-IF.

HCPs from different disciplines from the countries specified were approached by local recruiters to participate. Hospitals (including secondary and/or tertiary centers) were selected to provide national coverage within each country.

Eligible HCPs (including gastroenterologists, nutrition specialists, dieticians, nurses, surgeons, or any other country-specific professional) managing patients with SBS-IF and who had prescribed teduglutide to ≥5 patients with SBS-IF receiving PS were surveyed. HCPs were excluded if they had no experience managing patients with SBS-IF or no previous experience administering teduglutide treatment in this patient population.

### 2.2. Data Collection

The survey was completed voluntarily by HCPs between November 2022 and March 2023, and the survey explored the standard of care only for patients with SBS-IF indicated to receive teduglutide and not for patients with renal impairment. No patient-level information was provided.

The survey included screening questions (such as the caseload of patients with SBS-IF managed by each HCP) and collected demographic data from HCPs and data relating to their experience in managing adult patients with SBS-IF who were treated with teduglutide and (1) achieved PS independence, (2) maintained PS volume stability for ≥12 months (maximum teduglutide efficacy), and (3) discontinued teduglutide temporarily (treatment break). For patient groups 1 and 2, data collected included treatment patterns, criteria used to discontinue or adjust teduglutide dosing, strategies for discontinuation and reintroduction of teduglutide, and criteria for treatment success. Owing to the small proportion of HCPs who temporarily discontinued teduglutide, the results for patient management and outcomes in such patients were inconclusive and are not presented here.

### 2.3. Sample Size

Given that the study was descriptive, formal sample size calculations were not performed.

### 2.4. Statistical Analysis

Descriptive analyses were performed. All analyses were conducted for all respondents who were eligible for inclusion and who completed the survey. Continuous variables were described using mean with standard deviation (or medians and interquartile range [IQR]). Categorical variables were described using counts with percentages. Only HCPs completing the entire survey were included in this study, and therefore, there were no missing data. Owing to the small number of HCPs in Austria, data from Austria and Germany were pooled to preserve respondent anonymity.

### 2.5. Ethics

The study (protocol number TAK-633-4009, version 6.0) was conducted under the European Pharmaceutical Market Research Association (EphMRA) guidelines in all countries, ensuring that the respondents were pseudo-anonymized throughout. Methodological review was sought by a centralized institutional review board (IRB)/ethics committee, along with confirmation of there being no requirement for ethical review due to no direct patient involvement (Pearl IRB, Indianapolis, IN, USA; approval 24 October 2022) [36,37]. General Data Protection Regulation (GDPR)-compliant informed consent was obtained from all participating HCPs.

## 3. Results

### 3.1. Study Population

Overall, 70 HCPs, from six countries, who treat adult patients with SBS-IF and prescribe teduglutide completed the online survey, with the cohort distributed as follows: Austria (*n* = 3), Canada (*n* = 15), France (*n* = 14), Germany (*n* = 14), Italy (*n* = 10), and Spain (*n* = 14) (Table 1). No more than two HCPs were recruited from a single center. Most HCPs were gastroenterologists (84%) and had been practicing for more than 10 years (79%) in tertiary or academic settings (87%). Overall, the median (IQR) caseload of patients with SBS-IF that the HCPs reported managing was 17 (9–28). The median (IQR) caseload for patients treated with teduglutide and achieving PS independence or PS volume stability for ≥12 months was 5 (2–10) cases and 4 (2–6) cases, respectively (Table 1).

**Figure 1:**
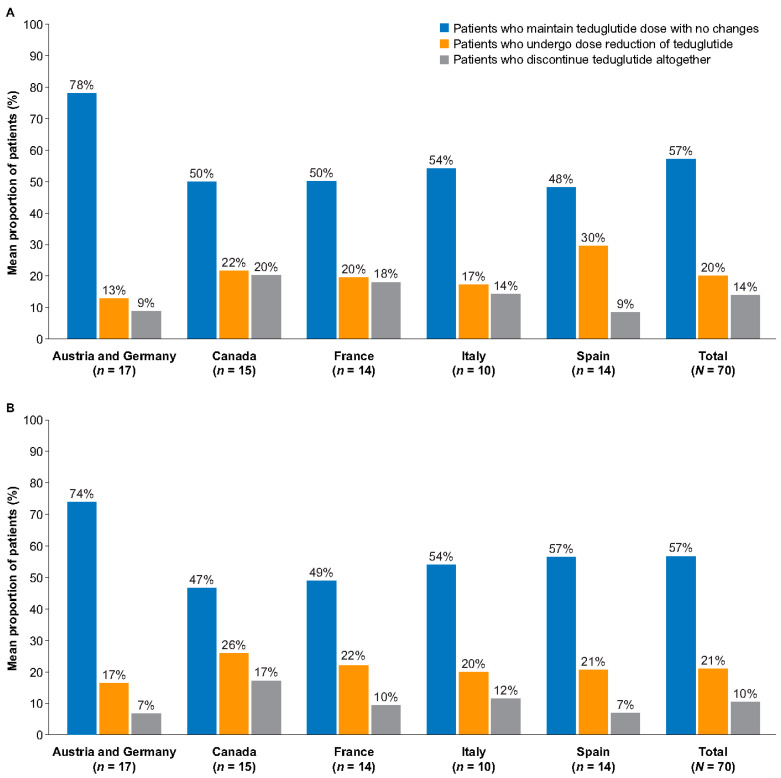
Teduglutide dose strategy for patients with SBS-IF who achieved PS independence (**A**) or PS volume stability for ≥12 months (**B**) after teduglutide treatment. IF, intestinal failure; PS, parenteral support; SBS, short bowel syndrome.

Nearly all HCPs (99%) reported managing patients with SBS-IF who had achieved PS independence and were receiving teduglutide at the standard dose, without changes, over 12 months of treatment (mean: 57% of patients [defined as the mean of the proportions of patients reported by all HCPs]; Table 2). Additionally, 71% of all HCPs reported managing patients who had undergone teduglutide dose reduction (either reduced frequency or dose; mean: 20% of patients), and 74% reported managing patients who had discontinued teduglutide treatment (mean: 14% of patients). There were differences regarding the willingness of HCPs to adopt a dose modification among the countries surveyed, with Austria and Germany having the lowest proportion of HCPs willing to reduce the teduglutide dose (Figure 1A,B).

HCPs estimated that patients who had undergone teduglutide dose reduction or discontinuation remained independent of PS for a median (IQR) of 12 (6–36) months and 12 (6–24) months, respectively. In total, 70% and 75% of HCPs managing patients who had undergone teduglutide dose reduction (*N* = 50) or discontinuation (*N* = 52), respectively, indicated they would reintroduce PS alone or in combination with teduglutide if a patient’s condition worsened (defined as an increased need for PS and a decrease in patient QoL) (Table S1). Of the 50 HCPs managing patients who underwent teduglutide dose reduction, 88% expected that these patients would remain PS-independent and not require PS reintroduction. Of the remaining 12% (6 HCPs), 4 indicated that ≤40% of patients would require PS reintroduction (Table S2). Of 52 HCPs managing patients who discontinued teduglutide altogether, 73% expected that these patients would remain PS-independent and not require PS reintroduction. Of the remaining 27% (14 HCPs), 11 indicated that ≤40% of patients would require PS reintroduction (Table S2).

Of the 52 HCPs managing patients who discontinued teduglutide, 81% stated that they would restart teduglutide alone or in combination with PS if a patient’s condition worsened (Table S1). Of these 52 HCPs, 42 answered questions related to what factors affect the decision to restart teduglutide in these patients. Of these 42 HCPs, 62% reported that there was no “threshold level” of PS (i.e., a standard increase in PS volume) required before teduglutide was reconsidered for reinitiation. For patients restarting teduglutide, 76% of HCPs would do so at the standard dose. Of HCPs who would restart teduglutide, 71% indicated that PS could be reduced over time, with the goal of achieving PS independence again, whereas the remaining 29% indicated that they would reduce PS but not aim for PS independence. More than 70% of HCPs indicated that negative clinical outcomes (79%) and patient treatment preferences and decreased patient QoL (74%) were the main factors affecting their decision to restart teduglutide in these patients. Finally, 52 HCPs expected that a mean of 46% of the patients who discontinued teduglutide were likely to restart teduglutide 3–12 months after discontinuation (Figure S1).

### 3.2. Patients with SBS-IF Maintaining PS Volume Stability for ≥12 Months (Maximum Teduglutide Efficacy)

Nearly all HCPs (97%) reported managing patients who had achieved PS volume stability for ≥12 months after teduglutide treatment and maintained the standard teduglutide dose without changes (mean: 57% of patients; Table 2). Additionally, 67% of all HCPs reported managing patients who had achieved PS volume stability and had undergone teduglutide dose reduction (reduced frequency or lower dose; mean: 21% of patients), and less than half (46%) of all HCPs reported managing patients who discontinued teduglutide after achieving PS volume stability (mean: 10% of patients) (Table 2).

As observed for patients who achieved PS independence, HCPs in Austria and Germany indicated limited experience regarding teduglutide dose modification (Figure 1A,B; orange bar).

Nearly all HCPs (98%, 46/47) managing patients who achieved PS volume stability and underwent teduglutide dose reduction reported that these patients typically remained at a stable PS volume, with no need for any increase. Of these HCPs, 26 indicated that the median (IQR) duration of PS volume stability in these patients was 12 (6–18) months. Almost all HCPs (>90%) reported that patients who achieved PS stability for ≥12 months typically had a ≥20% reduction in PS volume from the baseline (Figure S2). All HCPs managing patients who discontinued teduglutide after achieving PS volume stability (*N* = 32) indicated they would not consider restarting teduglutide in these patients if a patient’s condition worsened.

### 3.3. Clinical Monitoring

Overall, there was only a limited difference in monitoring frequency (follow-up every 6–16 weeks) between patients with SBS-IF who achieved PS independence and those who maintained PS volume stability for ≥12 months after teduglutide treatment. However, several country-specific differences within each patient population were noted. In France, Italy, and Spain, follow-up in patients who achieved PS independence and underwent teduglutide dose reduction occurred more frequently than in patients achieving PS independence who maintained a standard teduglutide dose or discontinued treatment (Figure S3A). In Austria, Germany, and Italy, follow-up in patients who achieved PS volume stability and were maintaining their teduglutide dose occurred less frequently than in patients who had undergone teduglutide dose reduction or discontinuation (Figure S3B).

**Figure 2:**
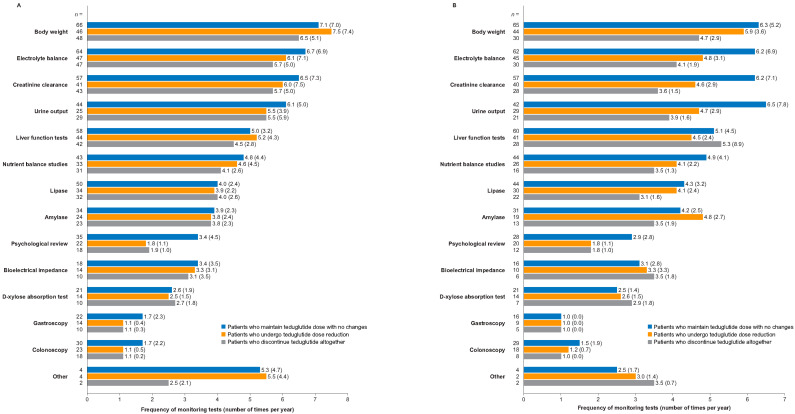
Frequency of monitoring tests for patients with SBS-IF who achieved PS independence (**A**) or PS volume stability for ≥12 months (**B**) after teduglutide treatment. Data to the left of the bars represent the number of patients. Data next to the bars represent mean (standard deviation). IF, intestinal failure; PS, parenteral support; SBS, short bowel syndrome.

**Figure 3:**
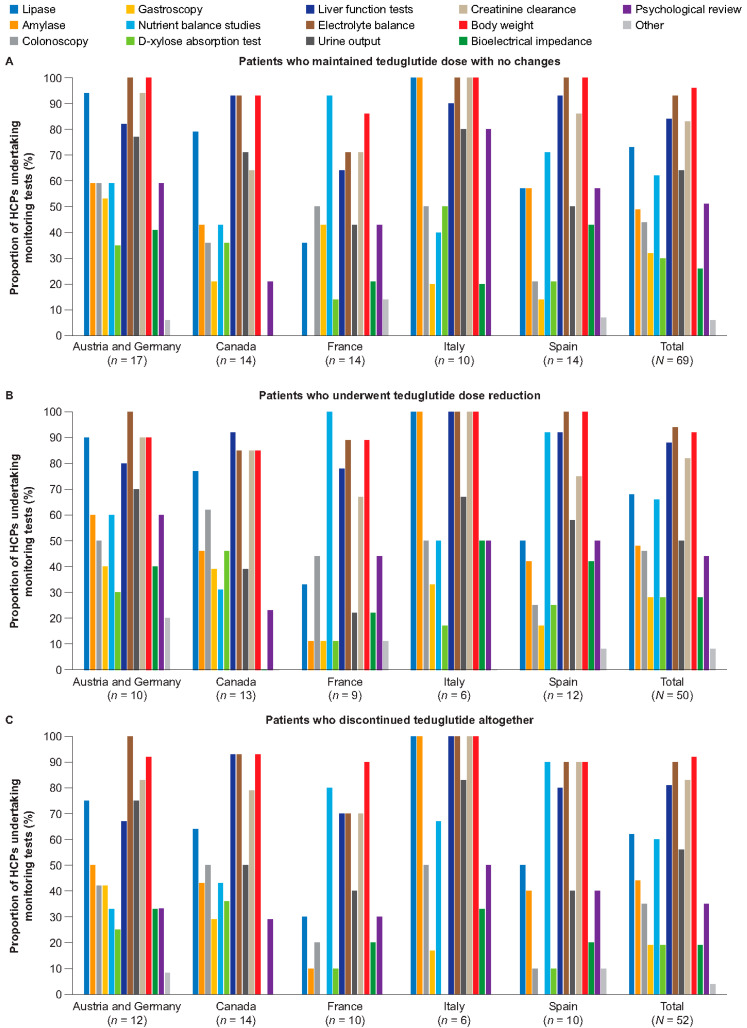
Tests performed to monitor patients with SBS-IF who achieved PS independence after initiating teduglutide treatment among patients who maintained teduglutide dose with no changes (**A**), who underwent teduglutide dose reduction (**B**), and who discontinued teduglutide (**C**). HCP, healthcare professional; IF, intestinal failure; PS, parenteral support; SBS, short bowel syndrome.

Similarly, there was little difference between the clinical measures HCPs used to monitor patients in either of the two groups. HCPs reported conducting the following: quarterly lipase, liver function, and creatinine clearance measurements, along with nutrient balance studies; twice-yearly bioelectrical impedance tests, D-xylose absorption tests, and psychological review; and annual testing of invasive procedures such as gastroscopy and colonoscopy, when indicated (Figure 2). Several country-specific differences in the clinical measures taken within each patient population were noted (Figure 3 and Figure S4).

## 4. Discussion

The clinical efficacy and safety of the standard teduglutide dose in adult patients with SBS-IF has been demonstrated in three clinical trials (STEPS, STEPS-2, and STEPS-3), as well as in real-world studies and a recent SLR [1,2,4,10,13,15,22,24,25,26,27,28,29,30]. These studies demonstrated that teduglutide can reduce PS volume and increase the likelihood of achieving PS independence [1,2,4,10,13,15,22,24,25,26,27,28,29,30]. However, only a few guidelines exist regarding the use of GLP-2 analog therapies and how these therapies might be tailored to meet patient needs and maximize efficacy. There is a need to understand teduglutide treatment practices among HCPs, given that GLP-2 analogs would be administered chronically for most patients with SBS-IF. This study is the first to describe the real-world and long-term HCP-reported treatment patterns and management of patients with SBS-IF who achieve PS independence or stable PS volume with teduglutide use.

In this study, almost all HCPs had experience in treating patients with teduglutide who had achieved PS independence or PS volume stability over 12 months. Across all countries studied, most patients receiving teduglutide maintained the standard teduglutide dose, without dose adjustment, as recommended by the label. However, when compared with the other surveyed HCPs, a higher proportion of Canadian and Spanish HCPs initiated dose adjustments. In contrast, HCPs in Austria and Germany were more conservative in making dose adjustments, likely because dose modification constitutes off-label use (except for patients with renal impairment), and no published clinical data are available to support dose modifications in patients with SBS-IF. Furthermore, teduglutide dose reduction after achieving response, without any other indication for reducing the applied dose, is not a common practice in countries where approval status and on-label use are associated with reimbursement (U.-F. Pape, personal communication).

The majority of HCPs managing patients who achieve PS independence and reduce or discontinue teduglutide reported that these patients remain independent of PS for a median of 12 months. However, a minority of HCPs indicated that a proportion of these patients would require PS reintroduction after teduglutide dose reduction or discontinuation. As this is not a common practice in all participating countries included in this survey, it is likely that PS would not be reinitiated for certain patients. In such cases without reinitiation of PS, treatment with teduglutide may help patients to maintain PS independence. Furthermore, stable long-term treatment with teduglutide (≥12 months) is associated with a reduction in PS volume requirements compared with baseline volumes, as has previously been demonstrated [4,13,15,24,25,26,27,28]. For patients who require PS reintroduction after achieving PS independence and discontinuing teduglutide, over three-quarters of HCPs indicated that they would restart teduglutide at the standard dose. This is not surprising, given that the mechanism of action suggests that teduglutide may require steady-state pharmacological levels to continuously enhance the proliferation of mucosal enterocytes beyond spontaneously occurring adaption, thereby enhancing their functional capacity [3,38]. In contrast, for those patients who maintained PS volume stability and discontinued teduglutide, no HCPs would consider restarting teduglutide. This was an unexpected observation which could suggest some potential misunderstanding or ambiguity in the survey process, patient teduglutide tolerability issues, or other patient factors leading to a desire to stop treatment. Our findings suggest that continuous teduglutide treatment is used to maintain PS independence, which is consistent with current recommendations and other articles which report an additional burden that can be associated with stopping treatment [17,20,33]. In Poland, where GLP-2 analogs are available only to participants in clinical trials [39], a real-world study of 13 patients demonstrated that 12 months after cessation of teduglutide, more than two-thirds of patients required a median increase of 44% (~1.67 L) in PN volume per week. This increased need for weekly PN treatment was seen at 84 months, 108 months, and 9 years after teduglutide cessation, suggesting that continued teduglutide treatment is required to maintain benefits [40]. In France, a study demonstrated that there is a substantial risk of sarcopenia and malnutrition after weaning off PS, and that teduglutide treatment contributes to metabolic stability in those patients requiring PS [41].

Long-term monitoring of the safety and effectiveness of teduglutide is required for patients with SBS-IF who wean off PS to identify potential complications associated with their disease and to quickly adapt to changes in PS volume [33,35]. In this study, there was an overwhelming consensus regarding the type and frequency of monitoring procedures employed across the different patient types and countries evaluated, although evidence-based monitoring study data do not exist. Given that this is the first real-world study to investigate monitoring practices beyond guidelines and expert recommendations, the evidence generated here serves to support such guidelines recommending close monitoring of patients with SBS-IF to adjust treatment patterns [35].

One of the strengths of this study is that the sample size (despite being small) represents a significant proportion of the total number of prescribers because the target population of HCPs using teduglutide in patients with SBS-IF is small and thus, largely generalizable within the countries surveyed. As is inherent with observational studies, this study has several limitations. The survey was not a validated measure and relied on HCP experience and perspectives of treatment for three key patient types within the countries surveyed and not on patient charts; hence, information bias might have occurred. In addition, the results of this study may not be generalizable to other countries in which there are different treatment practices, reimbursement patterns, and market-access strategies regarding the use of teduglutide. This survey should be repeated in future studies to test its reliability. Although a custom recruitment approach targeting HCPs at centers specializing in the treatment of SBS-IF, including teduglutide use, as per the European Society for Clinical Nutrition and Metabolism (ESPEN) guidelines, was employed to mitigate any selection bias [35], this may result in the underrepresentation of HCPs not operating at such sites. To maintain the anonymity of the small number of physicians from Austria, the responses from these participants were pooled with those from Germany, which may have impacted the results.

## 5. Conclusions

According to HCPs in this study, patients achieve PS independence or PS volume stability with teduglutide treatment, which is in line with the results from the current literature. However, some HCPs who manage patients maintaining PS independence after teduglutide reduction or discontinuation foresee that a proportion of these patients will require PS reintroduction. Therefore, these results suggest that it may be advisable to continue teduglutide treatment uninterrupted (i.e., no dose reduction or discontinuation) unless clinically indicated, but this requires confirmation in future studies.

## Figures and Tables

**Table 1:** HCP demographics.

	Country					
Characteristic	Austria and Germany (*n* = 17)	Canada (*n* = 15)	France (*n* = 14)	Italy (*n* = 10)	Spain (*n* = 14)	All HCPs (*n* = 70)
**Primary medical specialty, *n* (%)**						
Endocrinologist	0 (0)	0 (0)	0 (0)	0 (0)	5 (36)	5 (7)
Gastroenterologist	17 (100)	14 (93)	11 (79)	8 (80)	9 (64)	59 (84)
Nurse	0 (0)	0 (0)	0 (0)	0 (0)	0 (0)	0 (0)
Pharmacist	0 (0)	1 (7)	0 (0)	0 (0)	0 (0)	1 (1)
Surgeon	0 (0)	0 (0)	2 (14)	0 (0)	0 (0)	2 (3)
Nutrition specialist	0 (0)	0 (0)	1 (7)	0 (0)	0 (0)	1 (1)
Psychologist	0 (0)	0 (0)	0 (0)	0 (0)	0 (0)	0 (0)
Dietitian	0 (0)	0 (0)	0 (0)	2 (20)	0 (0)	2 (3)
Other	0 (0)	0 (0)	0 (0)	0 (0)	0 (0)	0 (0)
**Time practicing as a qualified specialist, years (%)**						
<5	0 (0)	0 (0)	0 (0)	0 (0)	0 (0)	0 (0)
5–10	3 (18)	2 (13)	3 (21)	3 (30)	4 (29)	15 (21)
11–15	2 (12)	6 (40)	5 (36)	5 (50)	5 (36)	23 (33)
16–20	8 (47)	3 (20)	3 (21)	1 (10)	3 (21)	18 (26)
≥21	4 (24)	4 (27)	3 (21)	1 (10)	2 (14)	14 (20)
**Primary setting, *n* (%)**						
Specialist treatment center	5 (29)	7 (47)	2 (14)	1 (10)	4 (29)	19 (27)
Other academic hospital	6 (35)	3 (20)	8 (57)	0 (0)	9 (64)	26 (37)
Other teaching hospital	5 (29)	2 (13)	2 (14)	6 (60)	1 (7)	16 (23)
Other nonteaching hospital	1 (6)	3 (20)	2 (14)	3 (30)	0 (0)	9 (13)
**HCP respondent caseload of patients with SBS-IF, median (IQR)**						
Adult patients with SBS-IF who achieved PS independence after initiation of teduglutide	4 (3–7)	5 (2–10)	7 (3–10)	2 (1–3)	6 (2–10)	5 (2–10)
Adult patients with SBS-IF who achieved PS volume stability after ≥12 months of teduglutide treatment	3 (2–6)	5 (2–10)	7 (3–10)	3 (2–4)	5 (3–5)	4 (2–6)
Total number of adult patients with SBS-IF	12 (9–23)	25 (10–30)	29 (19–30)	7 (5–9)	18 (10–23)	17 (9–28)

Owing to rounding, total(s) may not be equal to 100%. HCP, healthcare professional; IF, intestinal failure; IQR, interquartile range; PS, parenteral support; SBS, short bowel syndrome.

**Table 2:** Proportion of HCPs managing patients with short bowel syndrome and intestinal failure who achieved PS independence or PS volume stability after teduglutide treatment.

	Country					
Treatment Category	Austria and Germany (*n* = 17)	Canada (*n* = 15)	France (*n* = 14)	Italy (*n* = 10)	Spain (*n* = 14)	All HCPs (*n* = 70)
**HCPs managing patients who achieved PS independence, *n* (%)**						
Patients who maintained teduglutide dose, with no changes	17 (100)	14 (93)	14 (100)	10 (100)	14 (100)	69 (99)
Patients who underwent teduglutide dose reduction	10 (59)	13 (87)	9 (64)	6 (60)	12 (86)	50 (71)
Patients who discontinued teduglutide altogether	12 (71)	14 (93)	10 (71)	6 (60)	10 (71)	52 (74)
**HCPs managing patients who achieved PS volume stability for ≥12 months, *n* (%)**						
Patients who maintained teduglutide dose, with no changes	17 (100)	14 (93)	13 (93)	10 (100)	14 (100)	68 (97)
Patients who underwent teduglutide dose reduction	10 (59)	13 (87)	8 (57)	6 (60)	10 (71)	47 (67)
Patients who discontinued teduglutide altogether	8 (47)	11 (73)	4 (29)	4 (40)	5 (36)	32 (46)

HCP, healthcare professional; PS, parenteral support.

## Data Availability

Data sets supporting the results from this study are available from the corresponding author upon reasonable request. The data sets will be provided after deidentification, in compliance with applicable privacy laws, data protection, and requirements for consent and anonymization.

## Supplementary Materials

The following supporting information can be downloaded at: https://www.mdpi.com/article/10.3390/nu16213762/s1, HCP Survey. Table S1: Treatment measures implemented by HCPs if the condition of a patient with SBS-IF who achieved PS independence worsened after undergoing teduglutide dose reduction or discontinuation. Table S2: Proportion of patients with SBS-IF who achieved PS independence and will require PS reintroduction after teduglutide dose reduction or discontinuation. Figure S1: Mean proportion of patients with SBS-IF who achieved PS independence and are expected to restart their teduglutide prescription over time after teduglutide discontinuation. Figure S2: Average maximal volume reduction from baseline (%) in PS observed in adult patients with SBS-IF achieving PS stability with ≥12 months of teduglutide treatment. Figure S3: Frequency of clinical monitoring for patients with SBS-IF who achieved PS independence (A) or PS volume stability with ≥12 months (B) after teduglutide treatment. Figure S4: Tests performed to monitor patients with SBS-IF who achieved PS volume stability for ≥12 months after teduglutide treatment.
