# Supplementary material for: Management Patterns of Teduglutide Use in Short Bowel Syndrome: A Survey of 70 Healthcare Professionals
Source: Nutrients. 2024 Nov 1;16(21):3762. doi: 10.3390/nu16213762 (PMC11547446; doi:10.3390/nu16213762)
Supplement: Supplementary file 1 [file nutrients-16-03762-s001.zip › nutrients-3209675-supplementary.pdf]

Supplementary Material

HCP SURVEY

HCP SCREENER

Please answer the following questions to check your eligibility to take part in this study: please go to the next page .

<ASK ALL>

SQ1. In which country is your main practice located?

Please select one answer

1	O	Austria (AT)
2	O	France (FR)
3	O	Germany (DE)
4	O	Spain (ES)
5	O	Canada (CA)
6	O	Italy (IT)
7	O	Other <SCREEN OUT>

SQ2. Please indicate your primary medical specialty

Please select one answer

1	O	Gastroenterologist
2	O	Nutritional Specialist
3	O	Surgeon
4	O	Endocrinologist
5	O	Psychologist
6	O	Pharmacist
7	O	Nurse
8	O	Dietitian
9	O	Other <SCREEN OUT>

SQ3. How many years have you been practicing as a qualified [<INSERT SPECIALTY FROM S2>]?
Select one response only

1	O	Less than five years <SCREEN OUT>
2	O	5 to 10 years
3	O	11 to 15 years
4	O	16 to 20 years
5	O	21 or more years

SQ4: How many adult patients with SBS are you directly involved in discussions that inform treatment decisions?
Note: Please also include all patients managed via teleconsultations as well as those that you see in person
Please enter a number in each row below:

a	Total number of adult SBS patients	____________________
b	Adult SBS patients achieving independence from parenteral support (PS) after initiating teduglutide in a real-world setting	____________________
c	Adult SBS patients treated with teduglutide and with stable PS volume for at least 12 months	____________________
d	Adult SBS patients who initiate teduglutide and then temporarily discontinue [treatment break]	____________________

Thank you for completing the screening questions; you are invited to participate in this survey. This comprises of:

- An online survey (lasting no longer than <30> minutes), capturing your attitudes and experiences of managing adult patients with short bowel syndrome requiring parenteral nutritional support who are treated with teduglutide.

Every possible step is taken to ensure confidentiality; however, there is always a minimal risk remaining that confidentiality may be lost. Whilst you personally are unlikely to benefit directly from participating in the survey, your participation will lead to general knowledge about treating the condition/disease area.
Your participation is voluntary, and your alternative is not to participate. If you choose not to participate or leave the survey after agreeing to participate, there will be no penalty or loss of benefits to which you are otherwise entitled (based on your input into the survey at the point that you leave).
Click **here** to view our statement regarding data protection, privacy, invisible processing and ways to contact us.

Would you like to participate in this survey?

O	Yes
O	No <SCREEN OUT>

SECTION A: HCP DEMOGRAPHICS

AQ1: Which of the following best describes the primary setting in which you manage and advise adult SBS patients?

Select one response only

1	O	Specialist treatment center
2	O	Other academic hospital
3	O	Other teaching hospital
4	O	Other non-teaching hospital

SECTION B: MANAGEMENT OF ADULT SBS PATIENTS TREATED WITH TEDUGLUTIDE ACHIEVING INDEPENDENCE FROM PS

Within this section of the survey, please answer the questions thinking only about adult SBS patients achieving independence from parenteral support (PS) following initiation of teduglutide

BQ1: Do you see patients who use teduglutide in the following ways? If yes, in what proportion?
Select all that apply
Please enter numerical figure for percentage

1	Patients who maintain teduglutide dose with no changes	O	______%
2	Patients who undergo dose reduction of teduglutide (lower drug dose/reduced dose frequency)	O	______%
3	Patients who discontinue teduglutide altogether	O	______%

Thinking **ONLY** of those patients who maintain the standard teduglutide dose with no changes:

BQ2: How often do you assess these patients to monitor efficacy and tolerability of teduglutide and their need for PS?
Select one response only Please enter numerical figure

O	Every__weeks	OR	O	Every__months

BQ3: Which of the following tests do you undertake in order to monitor these patients? BQ3a: Which tests do you undertake?
BQ3b: How often do you undertake these tests? (number of times per year)
Select all that apply Enter numerical figure

	**Which tests do you undertake?**		**How often do you undertake test per year?**
1	Lipase	O	______________
2	Amylase	O	______________
3	Colonoscopy	O	______________
4	Gastroscopy	O	______________
5	Nutrient balance studies	O	______________
6	D-xylose absorption test	O	______________
7	Liver function tests	O	______________
8	Electrolyte balance	O	______________
9	Urine output	O	______________
10	Creatinine clearance	O	______________
11	Body weight	O	______________
12	Bioelectrical impedance	O	______________
13	Psychological review (counselling etc)	O	______________
14	Other (please specify)__	O	______________

Thinking now **ONLY** of those patients who have undergone a dose reduction of teduglutide:

BQ4: How often do you assess these patients to monitor efficacy and tolerability of teduglutide and their need for parenteral support?
Select one response only Please enter numerical figure

O	Every__weeks	OR	O	Every__months

BQ5: What dose of teduglutide is typically given to these patients?
Select one response only Please enter numerical figure

¡	Every day	_______ mg/kg
O	Every second day	
O	Every week	

BQ6: Which of the following tests do you undertake in order to monitor these patients? BQ6a: Which tests do you undertake?
BQ6b: How often do you undertake these tests? (number of times per year)
Select all that apply Enter numerical figure

	**Which tests do you undertake?**		**How often do you undertake test per year?**
1	Lipase	O	______________
2	Amylase	O	______________
3	Colonoscopy	O	______________
4	Gastroscopy	O	______________
5	Nutrient balance studies	O	______________
6	D-xylose absorption test	O	______________
7	Liver function tests	O	______________
8	Electrolyte balance	O	______________
9	Urine output	O	______________
10	Creatinine clearance	O	______________
11	Body weight	O	______________
12	Bioelectrical impedance	O	______________
13	Psychological review (counselling etc)	O	______________
14	Other, (please specify)__	O	______________

BQ7: Do these patients **typically** remain independent of PS?
Single response only

1	¡	Yes
2	O	No

BQ8: How long do these patients **typically** remain independent of PS?
Select one response only Please enter numerical figure

O	_______months	OR	O	_______years

BQ9: Typically, what proportion of these patients require a reintroduction of their PS?
Single response only

1	O	10-20%
2	O	20-30%
3	O	30-40%
4	O	50-60%
5	O	>60%

BQ10a: Of those who require PS reintroduction how is their parenteral support prescription **typically** managed?
Single response only

1	O	Restart PS at pre teduglutide volume
2	O	Gradual reintroduction of PS

BQ10b. Over what period does the gradual increase occur?
Select one response only Enter numerical figure

¡	_______Days	¡	_______Weeks

BQ11: Would you re-introduce parenteral support or increase teduglutide back to the original dose if the patient's performance worsened?
Single response only

1	¡	Re-introduce PS
2	O	Increase teduglutide back to the baseline dose
3	O	Both of the above

Thinking now **ONLY** of those patients who have discontinued their teduglutide treatment:

BQ12: How often do you assess these patients to monitor if there is a need for parenteral support?
Select one response only Please enter numerical figure

O	Every__weeks	OR	O	Every__months

BQ13: Which of the following tests do you undertake in order to monitor these patients? BQ13a: Which tests do you undertake?
BQ13b: How often do you undertake these tests? (number of times per year)
Select all that apply Enter numerical figure

	Which tests do you undertake?		How often do you undertake test per year?
1	Lipase	O	____________
2	Amylase	O	____________
3	Colonoscopy	O	____________
4	Gastroscopy	O	____________
5	Nutrient balance studies	O	____________
6	D-xylose absorption test	O	____________
7	Liver function tests	O	____________
8	Electrolyte balance	O	____________
9	Urine output	O	____________
10	Creatinine clearance	O	____________
11	Body weight	O	____________
12	Bioelectrical impedance	O	____________
13	Psychological review (counselling etc)	O	____________
14	Other, (please specify)__	O	____________

BQ14: Do these patients **typically** remain independent of PS?
Single response only

1	¡	Yes
2	O	No

BQ15: How long do these patients **typically** remain independent ofPS?
Single response only
Please enter numerical figure

O	_______months	OR	O	_______years

BQ16: Typically, what proportion of these patients require a reintroduction of their PS?
Single response only

1	O	10-20%
2	O	20-30%
3	O	30-40%
4	O	50-60%
5	O	>60%

BQ17a: Of those who require PS reintroduction how is their parenteral support prescription **typically** managed?
Single response only

1	O	Restart PS at pre teduglutide volume
2	O	Gradual reintroduction of PS

BQ17b. Over what period does the gradual increase occur?
Single response only Enter numerical figure

O	_______Days	O	_______Weeks

BQ18: Would you re-introduce parenteral support or restart teduglutide if the patient's performance worsened?
Single response only

1	O	Re-introduce PS
2	O	Restart teduglutide
3	O	Both of the above

BQ19: What factors affect a decision to restart teduglutide?
Select all that apply

1	Patient quality of life and preference	O
2	Clinical outcomes and assessments	O
3	Worsening of results of tests used to monitor patients	O
4	Other, (please specify) _	

BQ20: Is there a threshold level of parenteral support that would trigger the restart of teduglutide?
Single response only

1	O	Yes
2	O	No

BQ21: Where teduglutide is restarted in these patients, how is this done?
Single response only

1	O	Restart patient at standard dose used prior to decision to discontinue
2	O	Restart patient at reduced dose used prior to decision to discontinue

BQ22: Where teduglutide is restarted in these patients, what is the typical PS outcome?
Select one response only

1	O	PS can be reduced over time until independent
2	O	PS is reduced but not to the same extent

BQ23: What proportion of these patients would you expect to restart teduglutide in the following time frames post discontinuation?
Enter numerical figure

1	In under 3 months from teduglutide discontinuation	_______%
2	Between 3 and up to 6 months after teduglutide discontinuation	_______%
3	Between 6 and up to 12 months after teduglutide discontinuation	_______%
4	More than 12 months after teduglutide discontinuation	_______%
5	Not at all	_______%

BQ24: Which other healthcare professionals are involved in monitoring short bowel syndrome patients treated with teduglutide who have achieved independence from PS?

Select all that apply

1	O	Gastroenterologist
2	O	Nutritional Specialist
3	O	Surgeon
4	O	Endocrinologist
5	O	Psychologist
6	O	Pharmacist
7	O	Nurse
8	O	Dietitian
9	O	Other (please specify) __

SECTION C: MANAGEMENT OF ADULT SBS PATIENTS TREATED WITH TEDUGLUTIDE ACHIEVING STABLE PS VOLUME FOR AT LEAST 12 MONTHS

Within this section of the survey, please answer the questions thinking only about adult SBS patients treated with teduglutide with stable PS volume for at least 12 months

CQ1: What is the average maximal reduction in parenteral support that you observe in these patients?
Select one response only

1	O	<20% reduction in PS from baseline volume (volume at teduglutide initiation)
2	O	20-30% reduction in PS from baseline volume
3	O	40-50% reduction in PS from baseline volume
4	O	>50% reduction in PS from baseline volume
5	O	No reduction

CQ2: Do you see patients with a stable parenteral support volume who use teduglutide in the following ways? If yes, in what proportion?
Please select all that apply
Please enter numerical figure for percentage

1	Patients who maintain teduglutide dose with no changes	O	______%
2	Patients who undergo dose reduction of their teduglutide (lower drug dose/drug frequency)	O	______%
3	Patients who discontinue teduglutide altogether	O	______%

Thinking **ONLY** of those who maintain the standard teduglutide dose with no changes:

CQ3: How often do you review these patients to monitor efficacy and tolerability of teduglutide and their needs for parenteral nutritional support?
Select one response only Please enter numerical figure

O	Every _weeks	OR	O	Every _months

CQ4: Which of the following tests do you undertake in order to monitor these patients? CQ4a: Which tests do you undertake?
CQ4b: How often do you undertake these tests? (number of times per year)
Select all that apply Enter numerical figure

	**Which tests do you undertake?**		**How often do you undertake test per year?**
1	Lipase	O	____________
2	Amylase	O	____________
3	Colonoscopy	O	____________
4	Gastroscopy	O	____________
5	Nutrient balance studies	O	____________
6	D-xylose absorption test	O	____________
7	Liver function tests	O	____________
8	Electrolyte balance	O	____________
9	Urine output	O	____________
10	Creatinine clearance	O	____________
11	Body weight	O	____________
12	Bioelectrical impedance	O	____________
13	Psychological review (counselling etc)	O	____________
14	Other, (please specify) _	O	____________

Thinking now **ONLY** of those patients who have undergone a dose reduction of teduglutide:

CQ5: How often do you review these patients to monitor efficacy and tolerability of teduglutide and their needs for parenteral nutritional support?
Select one response only Please enter numerical figure

O	Every _weeks	OR	O	Every _months

CQ6: What dose of teduglutide is typically given to these patients?
Select one response only Please enter numerical figure:

O	Every day	_______ mg/kg
O	Every second day	
O	Every week	

CQ7: Which of the following tests do you undertake in order to monitor these patients? CQ7a: Which tests do you undertake?
CQ7b: How often do you undertake these tests? (number of times per year)
Select all that apply Enter numerical figure

	**Which tests do you undertake?**		**How often do you undertake test per year?**
1	Lipase	O	____________
2	Amylase	O	____________
3	Colonoscopy	O	____________
4	Gastroscopy	O	____________
5	Nutrient balance studies	O	____________
6	D-xylose absorption test	O	____________
7	Liver function tests	O	____________
8	Electrolyte balance	O	____________
9	Urine output	O	____________
10	Creatinine clearance	O	____________
11	Body weight	O	____________
12	Bioelectrical impedance	O	____________
13	Psychological review (counselling etc)	O	____________
14	Other, (please specify)__	O	____________

CQ8: Do these patients **typically** remain at a stable volume of parenteral support?
Single response only

1	O	Yes
2	O	No

CQ9: How long do these patients **typically** remain at a stable PS volume?
Select one response only Please enter numerical figure

O	_______months	OR	O	_______years

CQ10: Would you increase parenteral support volume or increase teduglutide back to the baseline dose if the patient's performance worsened?
Single response only

1	O	Increase PS volume
2	O	Increase teduglutide back to the higher dose
3	O	Both of the above

CQ11a: Of those patients requiring an increase in their PS, how is their parenteral support prescription **typically** managed?
Single response only

1	O	Return to PS at pre teduglutide volume
2	O	Gradual increase of PS

CQ11b. Over what period does the gradual increase occur?
Single response only Enter numerical figure

O	_______Days	O	_______Weeks

Thinking now of **ONLY** those patients who have discontinued their teduglutide treatment:

CQ12: How often do you review these patients to monitor their need for parenteral support?
Select one response only Please enter numerical figure

O	Every _weeks	OR	O	Every _months

CQ13: Which of the following tests do you undertake in order to monitor these patients? CQ13a: Which tests do you undertake?
CQ13b: How often do you undertake these tests? (number of times per year)
Select all that apply Enter numerical figure

	**Which tests do you undertake?**		**How often do you undertake test per year?**
1	Lipase	O	________
2	Amylase	O	________
3	Colonoscopy	O	________
4	Gastroscopy	O	________
5	Nutrient balance studies	O	________
6	D-xylose absorption test	O	________
7	Liver function tests	O	________
8	Electrolyte balance	O	________
9	Urine output	O	________
10	Creatinine clearance	O	________
11	Body weight	O	________
12	Bioelectrical impedance	O	________
13	Psychological review (counselling etc)	O	________
14	Other, (please specify)__	O	________

CQ14: Do you consider the option of restarting teduglutide in these patients?
Single response only

1	¡	Yes
2	O	No

Q type	SC	VAR		
Range				
Base	Only show to those who select code 3 [Patients who discontinue teduglutide altogether] in CQ2			
Logic				

CQ15: What factors affect a decision to restart teduglutide?
Select all that apply

1	Resolution of issues associated with teduglutide tolerability	O
2	Completion of episode of care that was the reason for teduglutide discontinuation e.g. surgery or other medical procedures completed	O
3	Patient preference	O
4	Change in patient's bodyweight	O
5	Desire to maintain reduced PS volume	O

CQ16: Is there a threshold level of parenteral support that would trigger the restart of teduglutide?
Single response only

1	¡	Yes
2	O	No

CQ17: Where teduglutide is restarted in these patients, how is this done?
Single response only

1	O	Restart patient at standard dose used prior to decision to discontinue
2	O	Restart patient at reduced dose used prior to decision to discontinue

CQ18: Where teduglutide is restarted, what is the typical outcome?
Select one response only

1	¡	PS can be reduced over time until at stable volume
2	O	PS is reduced but not to the same extent

CQ19: What proportion of these patients would you expect to restart teduglutide in the following time frames post discontinuation?

Enter numerical answers

1	In under 3 months from teduglutide discontinuation	_______%
2	Between 3 and up to 6 months after teduglutide discontinuation	_______%
3	Between 6 and up to 12 months after teduglutide discontinuation	_______%
4	More than 12 months after teduglutide discontinuation	_______%
5	Not at all	_______%

CQ20: Which other healthcare professionals are involved in monitoring adult short bowel syndrome patients who are treated with teduglutide and have had a stable PS volume for at least 12 months?

Select all that apply

1	O	Gastroenterologist
2	O	Nutritional Specialist
3	O	Surgeon
4	O	Endocrinolgist
5	O	Psychologist
6	O	Pharmacist
7	O	Nurse
8	O	Dietician
9	O	Other (please specify) __

SECTION D: MANAGEMENT OF ADULT SBS PATIENTS WHO TEMPORARILY STOP TEDUGLUTIDE

Within this section of the survey, please answer the questions thinking only about adult SBS patients who initiated teduglutide treatment and then temporarily discontinued [treatment break for reasons such as adverse events, patient choice, patient stability] EXCLUDING those discussed previously in this survey

DQ1: What proportion of SBS patients (excluding those already discussed in sections B and C of the survey) would you estimate temporarily stop teduglutide for any reason?

Please enter numerical figure as percentage proportion

_________________________ %

DQ2: What proportion of these patients would you estimate the reason to be related to the following:

Please enter numerical figure as percentage proportion

Chronic renal insufficiency	_______%
Medical care or procedures (e.g., surgery)]	_______%
Other comorbidities	_______%
Patient employment status	_______%
Clinical outcomes	_______%
Patient preference and quality of life	_______%
Other (please specify) _	_______%

DQ3: What is the average maximal reduction in parental support that you observe in these patients prior to the decision to temporarily stop teduglutide?

Select one response only

1	¡	<20% reduction in PS from baseline volume (volume at teduglutide initiation)
2	O	20-30% reduction in PS from baseline volume
3	O	40-50% reduction in PS from baseline volume
4	O	>50% reduction in PS from baseline volume
5	O	No reduction

DQ4: For patients who stop teduglutide treatment, what is the usual process for treatment withdrawal?
Select one response only

1	O	Immediate discontinuation
2	O	Dose reduction (lower drug dose/drug frequency)

Thinking **ONLY** of those patients on a treatment break who maintain a stable PS volume: DQ5: What proportion of patients remain at a stable PS volume?
Please enter numerical figure as percentage proportion

________________________ %

DQ6: How often do you review these patients to monitor their need for parenteral support?
Please enter numerical figure

O	Every _weeks	OR	O	Every _months

DQ7: Which of the following tests do you undertake in order to monitor these patients? DQ7a: Which tests do you undertake?
DQ7b: How often do you undertake these tests? (number of times per year)

Select all that apply Enter numerical figure

	**Which tests do you undertake?**		**How often do you undertake test per year?**
1	Lipase	O	______________
2	Amylase	O	______________
3	Colonoscopy	O	______________
4	Gastroscopy	O	______________
5	Nutrient balance studies	O	______________
6	D-xylose absorption test	O	______________
7	Liver function tests	O	______________
8	Electrolyte balance	O	______________
9	Urine output	O	______________
10	Creatinine clearance	O	______________
11	Body weight	O	______________
12	Bioelectrical impedance	O	______________
13	Psychological review (counselling etc)	O	______________
14	Other, (please specify)	O	______________

Thinking now **ONLY** of those on a treatment break who have an increased need for parenteral support:

DQ8: For patients who stop teduglutide treatment what proportion need to increase their PS volume?
Please enter numerical figure

________________________ %

DQ9. What are the factors that affect the decision to increase PS?
Select all that apply

1	Patient quality of life and preference	O
2	Clinical outcomes and assessments	O
3	Worsening of results of tests used to monitor patients	O
4	Other (please specify) _	O

DQ10: How is parenteral support typically managed in these patients?
Select one response only

1	O	Watch and wait approach to changes in PS volume
2	O	Immediate increase in PS volume to baseline level
3	O	Gradual increase in PS volume

DQ11. What proportion of patients maintain the following reduction from their baseline volume?
Please enter percentage figure for each row.

1	<20% reduction in PS compared to baseline volume (volume at initial introduction of teduglutide)	_______%
2	20-30% reduction in PS compared to baseline volume	_______%
3	31-40% reduction in PS compared to baseline volume	_______%
4	41-50% reduction in PS compared to baseline volume	_______%
5	>50% reduction in PS compared to baseline volume	_______%

DQ12: How often do you review these patients to monitor their need for parenteral support?
Please enter numerical figure

O	Every _weeks	OR	O	Every _months

DQ13: Which of the following tests do you undertake in order to monitor these patients? DQ13a: Which tests do you undertake?
DQ13b: How often do you undertake these tests? (number of times per year)
Select all that apply Enter numerical figure

	**Which tests do you undertake?**		**How often do you undertake test per year?**
1	Lipase	O	___________
2	Amylase	O	___________
3	Colonoscopy	O	___________
4	Gastroscopy	O	___________
5	Nutrient balance studies	O	___________
6	D-xylose absorption test	O	___________
7	Liver function tests	O	___________
8	Electrolyte balance	O	___________
9	Urine output	O	___________
10	Creatinine clearance	O	___________
11	Body weight	O	___________
12	Bioelectrical impedance	O	___________
13	Psychological review (counselling etc)	O	___________
14	Other, (please specify)__	O	___________

DQ14: Do you consider the option of restarting teduglutide in these patients?
Single response only

1	O	Yes
2	O	No

DQ15a: What factors affect a decision to restart teduglutide in these patients?

Select all that apply

1	Resolution of issues associated with teduglutide tolerability	O
2	Completion of episode of care that was the reason for teduglutide discontinuation e.g. surgery or other medical procedures completed	O
3	Patient preference	O
4	Change in patient's bodyweight	O
5	Desire to maintain reduced PS volume	O

DQ15b: Is there a threshold level of parenteral support that would trigger the restart of teduglutide in these patients?

Single response only

1	¡	Yes
2	O	No

DQ15c: Are there instances where you would not re-introduce teduglutide in these patients?

Select all that apply

1	O	Patient had a previous bad experience with teduglutide
2	O	Patient has chronic renal insufficiency
3	O	Patient has lack of reimbursed access to teduglutide
4	O	Lack of efficacy of teduglutide in patient

DQ16: Where teduglutide is restarted in these patients, how is this done?

Single response only

1	O	Restart patient at standard dose used prior to decision to discontinue
2	O	Restart patient at reduced dose used prior to decision to discontinue

DQ17: Where teduglutide is restarted, what is the typical PS outcome?

Select one response only

1	O	PS can be reduced over time until at stable volume
2	O	PS is reduced but not to the same extent

DQ18: What proportion of these patients would you expect to restart teduglutide in the following timeframes post-discontinuation?

Please enter percentage figure for each row

1	In under 3 months from teduglutide discontinuation	_______%
2	Between 3 and up to 6 months after teduglutide discontinuation	_______%
3	Between 6 and up to 12 months after teduglutide discontinuation	_______%
4	More than 12 months after teduglutide discontinuation	_______%
5	Not at all	_______%

DQ19: Which other healthcare professionals are involved in monitoring short bowel syndrome patients who are having a treatment break from teduglutide?

Select all that apply

1	O	Gastroenterologist
2	O	Nutritional Specialist
3	O	Surgeon
4	O	Endocrinologist
5	O	Psychologist
6	O	Pharmacist
7	O	Nurse
8	O	Dietitian
9	O	Other (please specify) __

END OF SURVEY

Thank you for participating in this study gathering information on real-world treatment patterns in adult short bowel syndrome patients treated with teduglutide.

This survey was funded by Takeda Pharmaceuticals.

Table S1 Treatment measures implemented by HCPs if the condition of a patient with SBS-IF who achieved PS independence worsened after undergoing teduglutide dose reduction or discontinuation.

Treatment strategy	Patients who underwent teduglutide dose reduction (*n* = 50)	Patients who discontinued teduglutide altogether (*n* = 52)
**Reintroduce PS, *n* (%)[a]**	11 (22)	10 (19)
**Increase teduglutide back to baseline dose, *n* (%)[a]**	15 (30)	13 (25)
**Undergo both measures, *n* (%)[a]**	24 (48)	29 (56)

[a]Data shown are the proportion of HCPs who would implement each treatment strategy.
HCP, healthcare professional; IF, intestinal failure; PS, parenteral support; SBS, short bowel syndrome.

Table S2 Proportion of patients with SBS-IF who achieved PS independence and will require PS reintroduction after teduglutide dose reduction or discontinuation.

Proportion of patients	HCPs managing patients who underwent teduglutide dose reduction, *n* (%) (*n* = 6)	HCPs managing patients who discontinued teduglutide altogether, *n* (%) (*n* = 14)
**10–20%**	2 (33)	1 (7)
**20–30%**	0 (0)	2 (14)
**30–40%**	2 (33)	8 (57)
**40–50%**	0 (0)	0 (0)
**50–60%**	1 (17)	2 (14)
**>60%**	1 (17)	1 (7)

HCP, healthcare professional; IF, intestinal failure; PS, parenteral support; SBS, short bowel syndrome.

Figure S1 Mean proportion of patients with SBS-IF who achieved PS independence and are expected to restart their teduglutide prescription over time after teduglutide discontinuation.

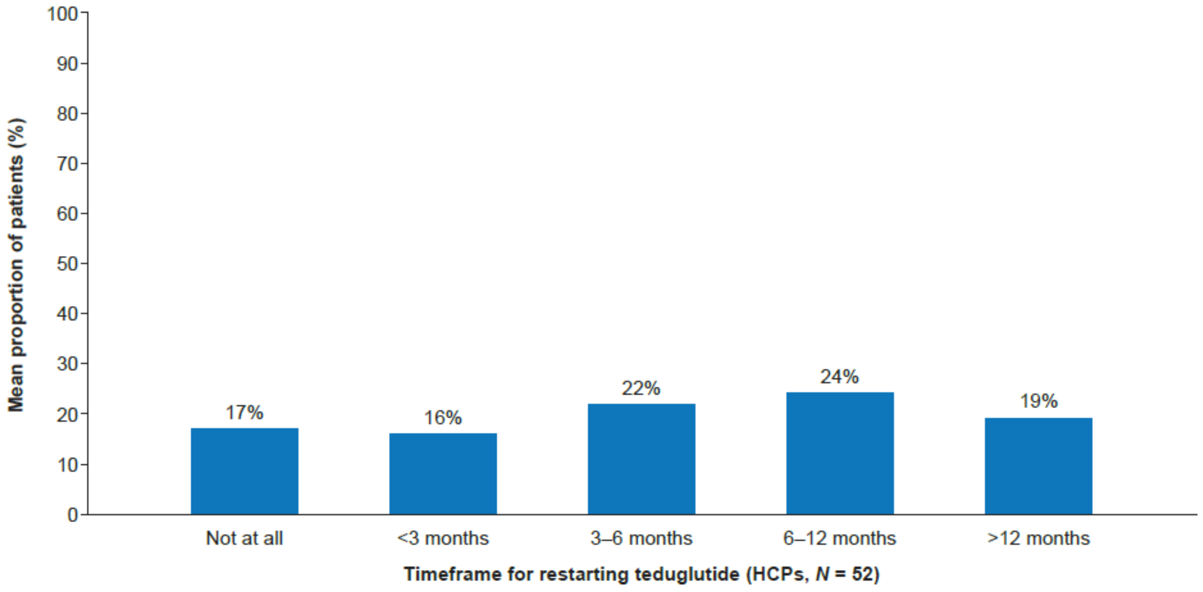

HCP, healthcare professional; IF, intestinal failure; PS, parenteral support; SBS, short bowel syndrome.

Figure S2 Average maximal volume reduction from baseline (%) in PS observed in adult patients with SBS-IF achieving PS stability for ≥12 months of teduglutide treatment.

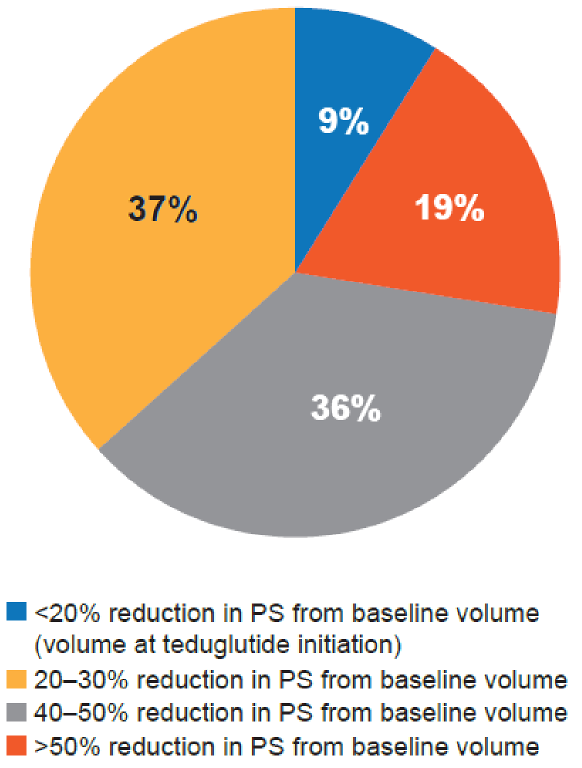

Owing to rounding, total(s) may not be equal to 100%.
Every patient with SBS-IF undergoing teduglutide treatment observed some degree of reduction in PS from baseline.
IF, intestinal failure; PS, parenteral support; SBS, short bowel syndrome.

Figure S3 Frequency of clinical monitoring for patients with SBS-IF who achieved PS independence (A) or PS volume stability for ≥12 months (B) after teduglutide treatment.

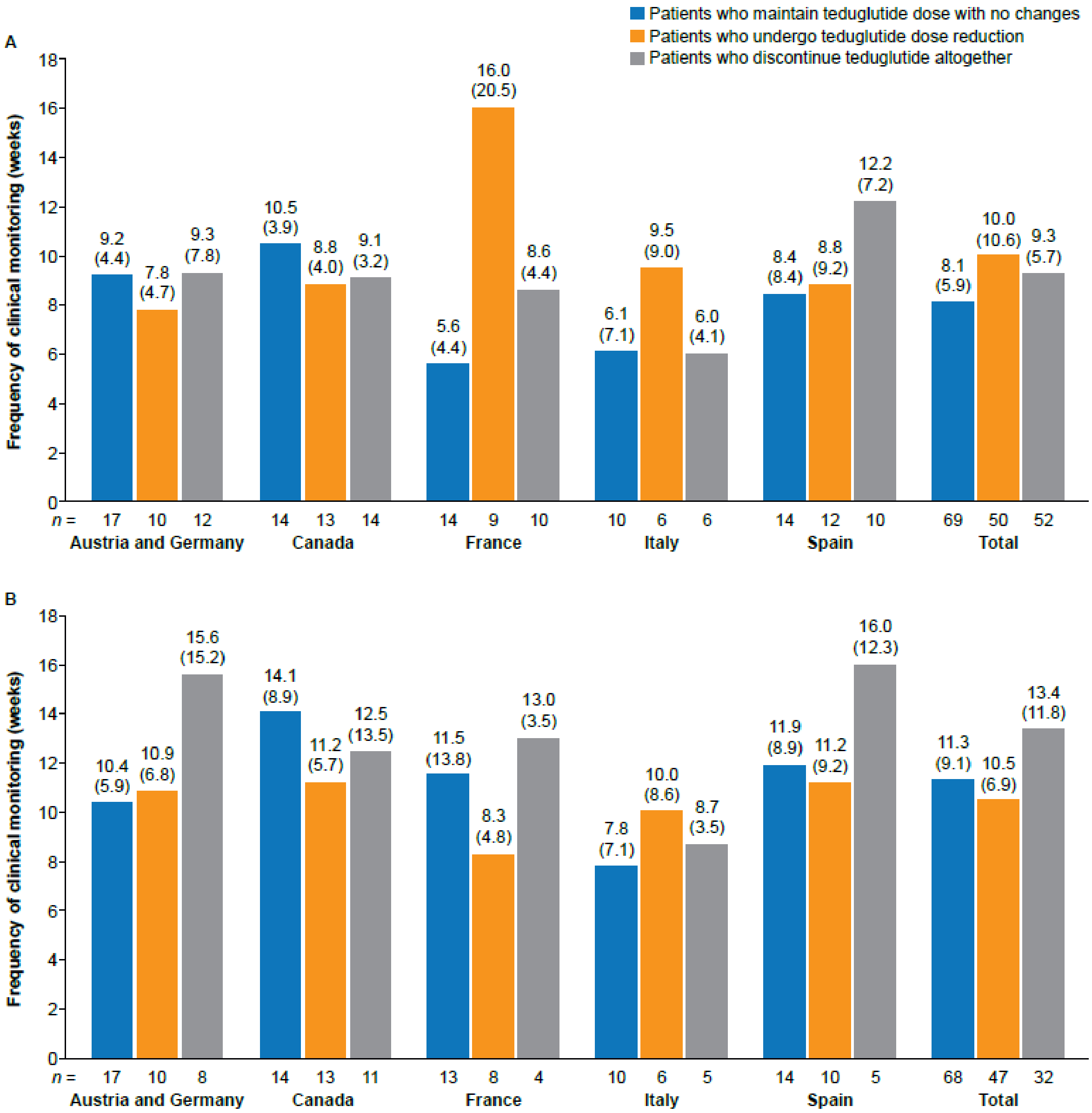

Data above bars represent mean (standard deviation). Data below bars represent the number of patients.
IF, intestinal failure; PS, parenteral support; SBS, short bowel syndrome.

Figure S4 Tests performed to monitor patients with SBS-IF who achieved PS volume stability for ≥12 months after teduglutide treatment.

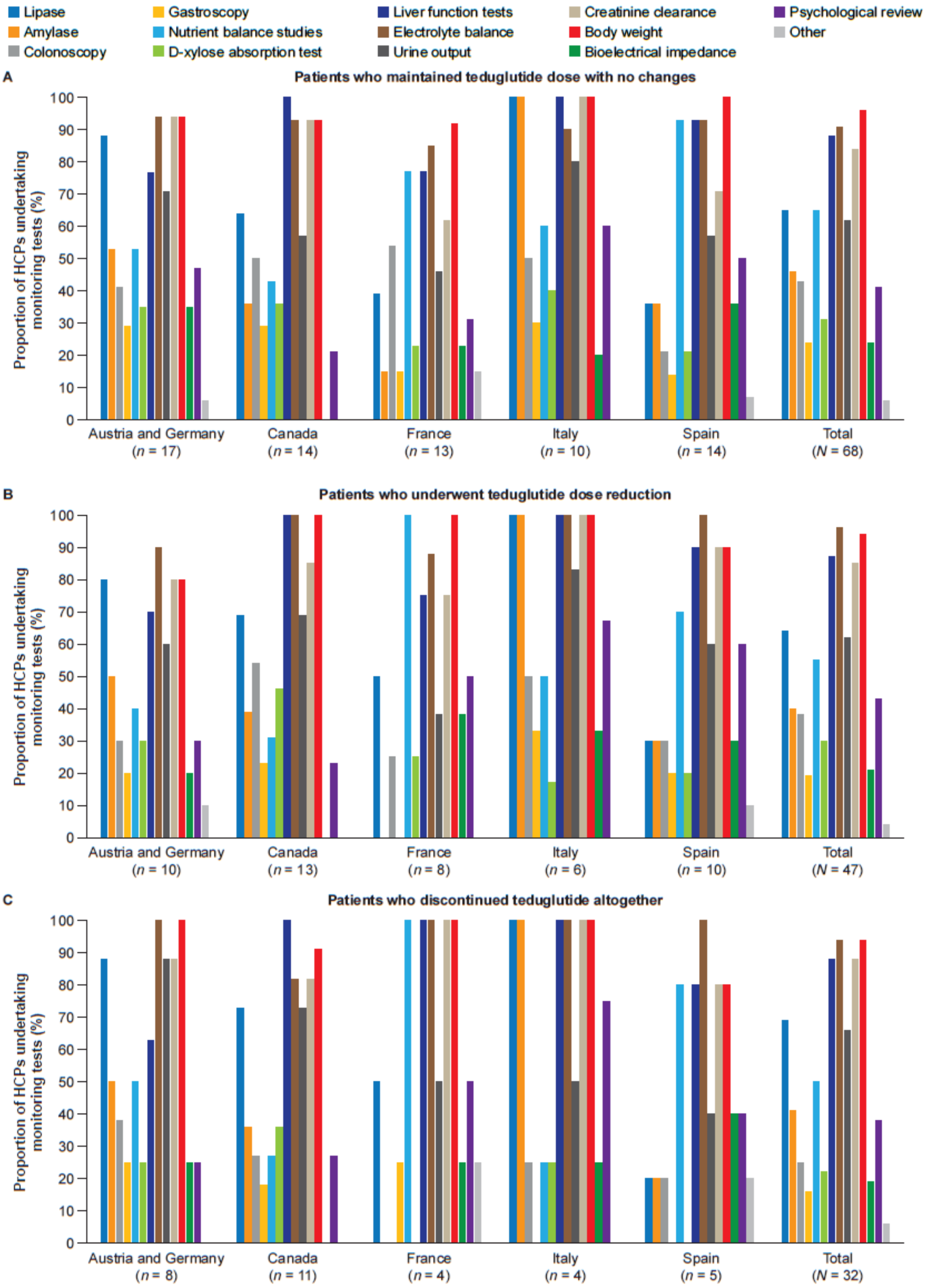

HCP, healthcare professional; IF, intestinal failure; PS, parenteral support; SBS, short bowel syndrome.
